# Supplementary material for: Telomere-to-telomere human DNA replication timing profiles
Source: Sci Rep. 2022 Jun 10;12:9560. doi: 10.1038/s41598-022-13638-8 (PMC9187705; doi:10.1038/s41598-022-13638-8)
Supplement: Supplementary file 1 — Supplementary Information. [file 41598_2022_13638_MOESM1_ESM.pdf]

Supplementary Information

Telomere-to-telomere human DNA replication timing profiles

Dashiell J. Massey, Amnon Koren

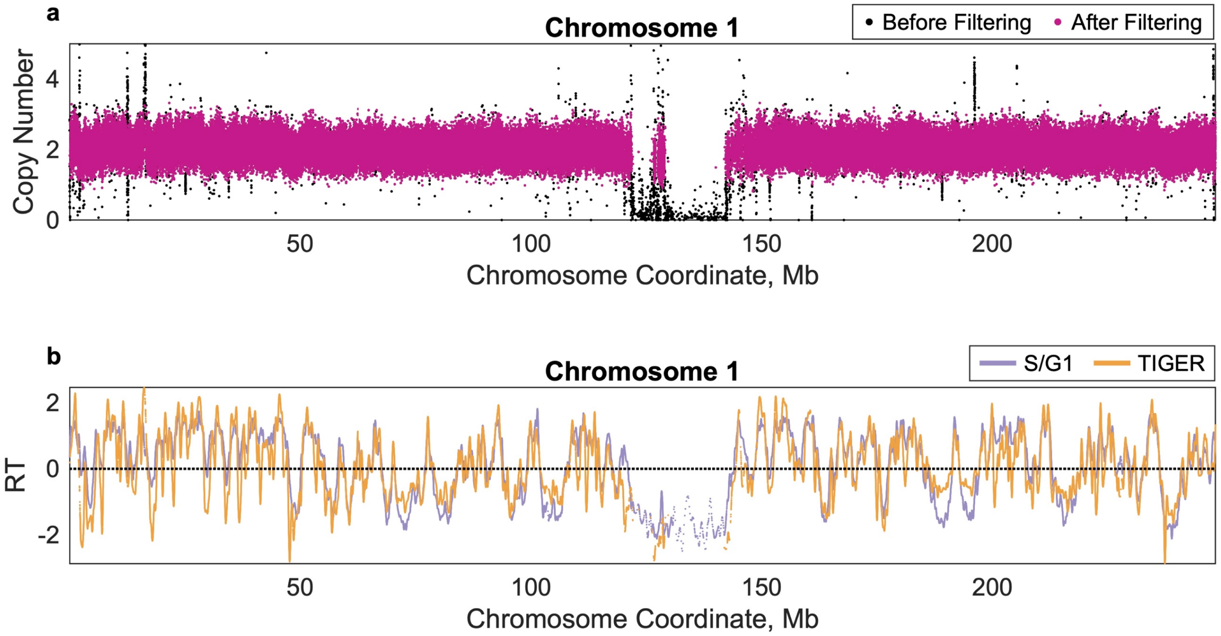

Supplementary Figure 1. **Replication timing analysis of highly repetitive regions requires a G_1-phase control sample. a** Mappability and GC-content corrected read counts for an asynchronous population of GM12878 cells before (black) and after (pink) filtering regions with abnormal copy-number estimates. The low coverage in the centromeric region is inadequately corrected even after accounting for these sequencing biases using TIGER[1]. The TIGER pipeline defines variable-size uniform-coverage genomic windows based on mappability biases of a given reference genome and performs library-specific GC-bias correction. **b** Similar replication timing profiles are obtained in non-repetitive regions of the genome between the S/G_1 and TIGER methods.

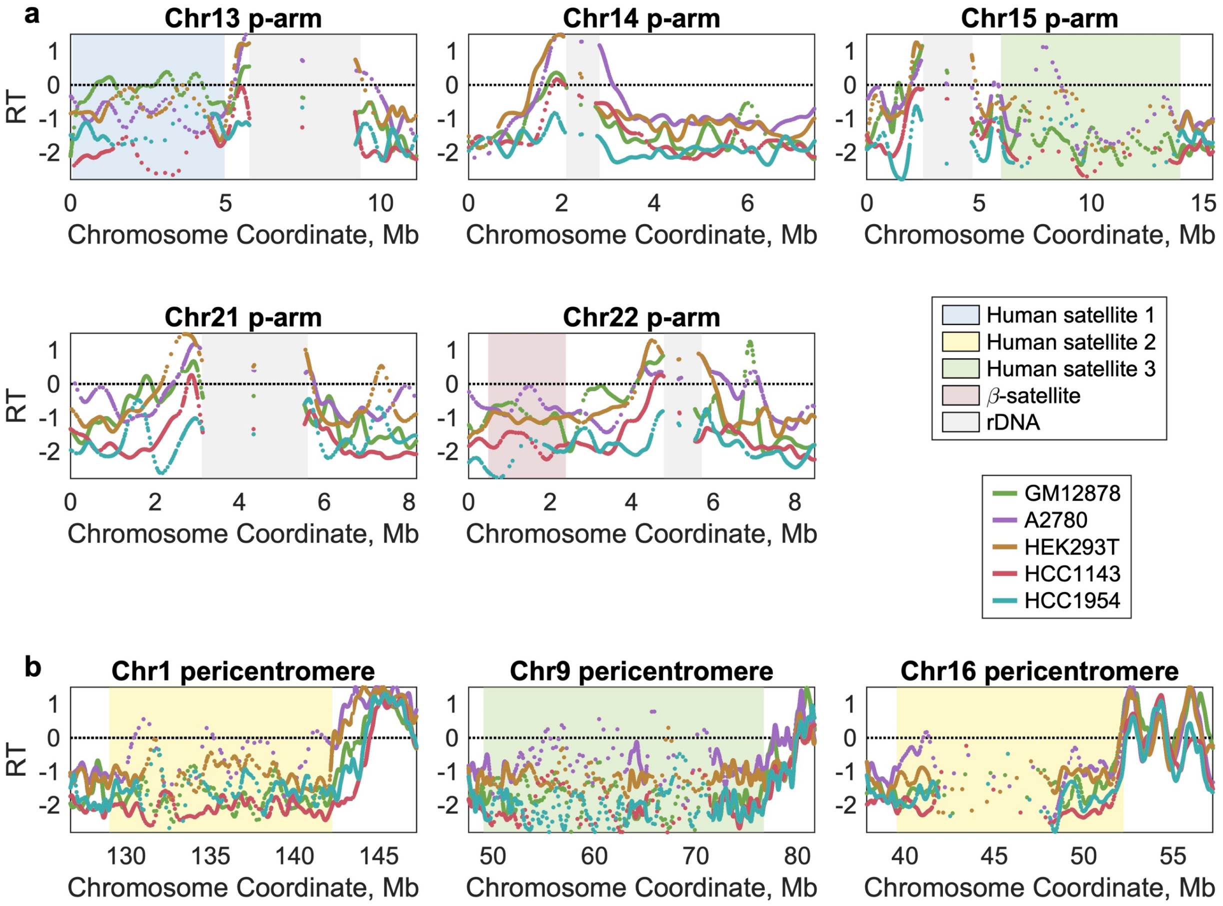

Supplementary Figure 2. **Replication timing (RT) of previously unresolved regions of the human genome for five cell lines.** Compare to Figure 2a,b.

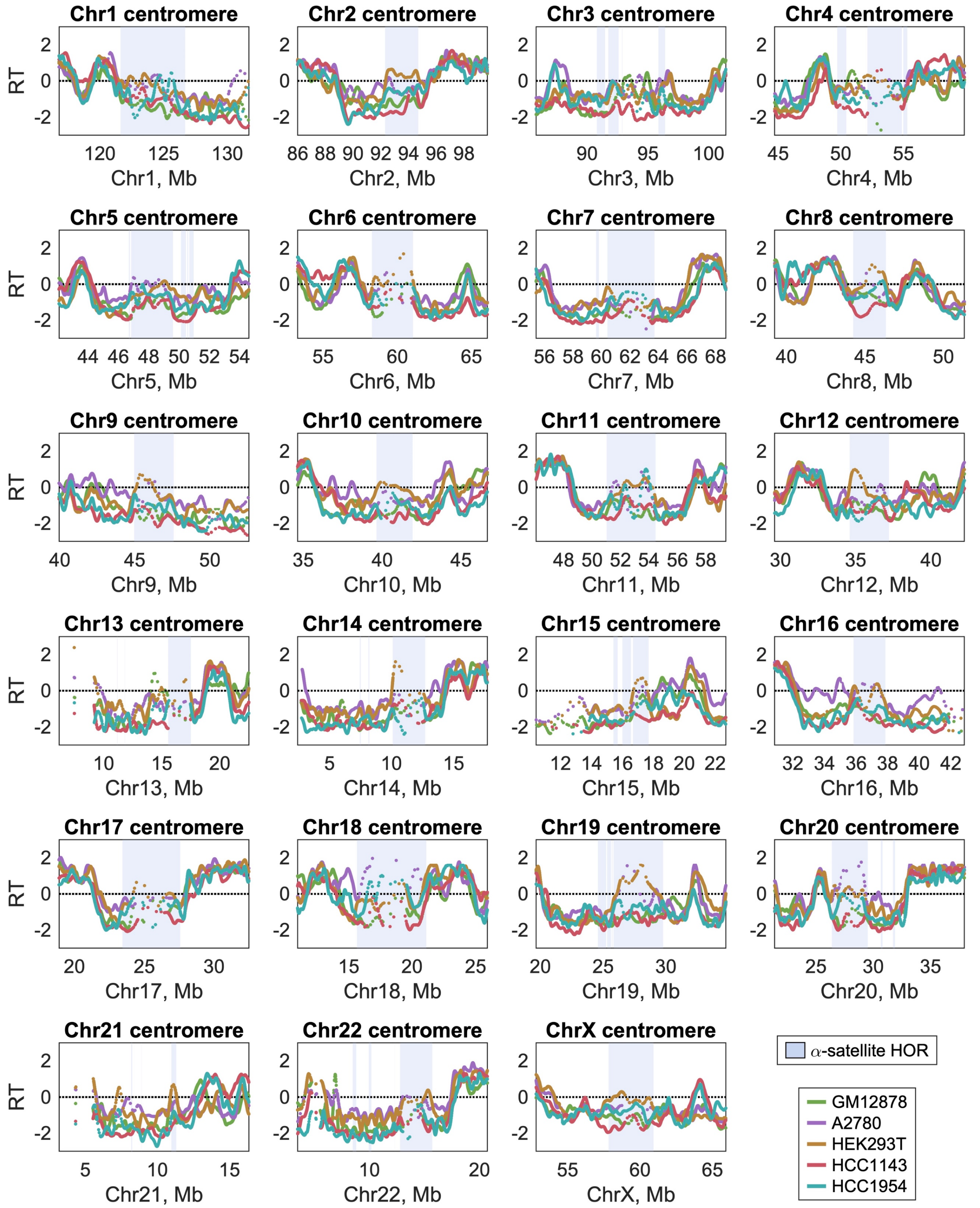

Supplementary Figure 3.**Centromere replication timing (RT) of all human autosomes and chromosome X for five cell lines.** Compare to Figure 3.

References

1 Koren, A., Massey, D. J. & Bracci, A. N. TIGER: inferring DNA replication timing from whole-genome sequence data. *Bioinformatics*, doi:10.1093/bioinformatics/btab166 (2021).
